# A possible association between early life factors and burden of functional bowel symptoms in adulthood

**DOI:** 10.1080/02813432.2021.2004823

**Published:** 2021-11-22

**Authors:** Johanna Wennerberg, Shantanu Sharma, Peter M. Nilsson, Bodil Ohlsson

**Affiliations:** a Department of Clinical Sciences, Lund University, Lund, Sweden; b Department of Internal Medicine, Skåne University Hospital, Malmö, Sweden

**Keywords:** Birth weight, early life factors, epidemiology, functional bowel symptoms, gestational age

## Abstract

**Objective:**

The studies of early life factors and development of functional bowel diseases show inconsistent results. We therefore examined associations between certain early life factors and functional bowel symptoms in adulthood.

**Design:**

Population-based cross-sectional study.

**Setting:**

Weight and height were measured and questionnaires were completed at the time point of enrollment in MOS.

**Subjects:**

1013 participants in the Malmö Offspring Study (MOS) without organic bowel disease with data available from the Swedish Medical Birth Registry.

**Main outcome measures:**

Associations were calculated between gestational age, birth weight, small-for-gestational-age and Apgar score from the Birth Registry, and symptoms according to the visual analog scale for irritable bowel syndrome (VAS-IBS) (abdominal pain, diarrhea, constipation, bloating and flatulence, vomiting and nausea, and symptoms’ influence on daily life) or self-reported IBS using logistic regression.

**Results:**

In all, 253 (25.0%) participants reported bowel symptoms during the past 2 weeks and 179 (17.7%) self-reported IBS; conditions which were strongly associated (*p* < 0.001). Female sex and chronic stress were two independent factors more common among participants with bowel symptoms compared with asymptomatic participants (*p* < 0.001). Early life factors were not associated with presence of overall bowel symptoms (*p* = 0.080), any specific bowel symptoms or self-reported IBS. Lower birth weight (*p* = 0.038) and being born small for gestational age (*p* = 0.043) were associated with severe influence of intestinal symptoms on daily life in adulthood.

**Conclusions:**

Lower birth weight and small for gestational age are not associated with the presence of overall bowel symptoms but with more pronounced influence of such symptoms on daily adult life.

**Key points**
- Lower gestational age tended to be associated with functional bowel symptoms in adulthood.
- Lower birth weight and being small for gestational age are associated with increased negative influences of symptoms on daily life in adulthood.
- Patients born preterm or with low birth weights may be at increased risk to develop functional bowel symptoms later in life.

## Introduction

The most common functional bowel disorder (FBD) is irritable bowel syndrome (IBS), characterized by abdominal pain and altered bowel habits in the absence of organic abnormalities [1]. The accumulated prevalence of IBS globally is around 11% [2]. Consequently, IBS adds a significant health economic burden to both society and patients [3]. Female sex, young age, lower physical activity and abdominal obesity are risk factors for IBS [2]. The levels of anxiety and depression are generally higher in IBS patients than in healthy controls [4], and psychological stress and stressful events are risk factors for the disease [2,5].

Different explanations contributing to the pathogenesis of FBD have been proposed, such as irregularities in the brain–gut interaction, bowel dysmotility, dysbiosis, enteric infections, immune activation, and stress [2]. Visceral hypersensitivity is a shared trait in most IBS patients, where both central and peripheral factors may contribute to the negatively altered sensory sensations [6].

The perinatal period is defined by the World Health Organization as starting in week 22 of gestation until 1 week after birth [7]. Preterm birth is defined as born before 37 completed weeks of pregnancy [8]. The amount of premature births is increasing, and for the babies that survive, there is an increased risk of disability and neuropsychological disorders [9]. On the other hand, a Swedish nationwide register-based cohort study found preterm birth to be associated with a decreased risk for IBS among young adults [10]. However, since the maturation of a fetus’ different organ systems occur at unique paces (time periods), a difference of one gestational week could lead to significantly different future health problems [8].

Low birth weight (LBW) (<2500 g) is associated with a variety of negative consequences [9,11]. Lower birth weight, although within the normal range, has been observed to be a risk factor for IBS [12,13]. The matching between birth weight and gestational age is defined as small for gestational age (SGA) when the birth weight in relation to gestational age is below the 10th percentile of the population distribution, but large for gestational age (LGA) when the birth weight is above the 90th percentile [14]. SGA carries higher risks of minor cognitive deficits and metabolic syndrome in adulthood [15]. Apgar score renders a quick status report regarding the newborn child’s well-being [16]. Studies have suggested that low Apgar score is also associated with an increased risk for pediatric bowel morbidity [17].

Our hypothesis was that adverse early life factors could negatively affect the maturation of the bowel system and contribute to the development of functional bowel symptoms. The aim of the present observational study was to investigate possible associations between early life factors and the development of functional bowel symptoms and self-reported IBS in adult life.

## Material and methods

The study was performed in accordance with the Helsinki declaration and approved by the Regional Ethics Review Board, Lund University (2012/594, date of approval 05/12/2012). All participants gave their written, informed consent before entering the study.

### Study design

**Figure 1.**
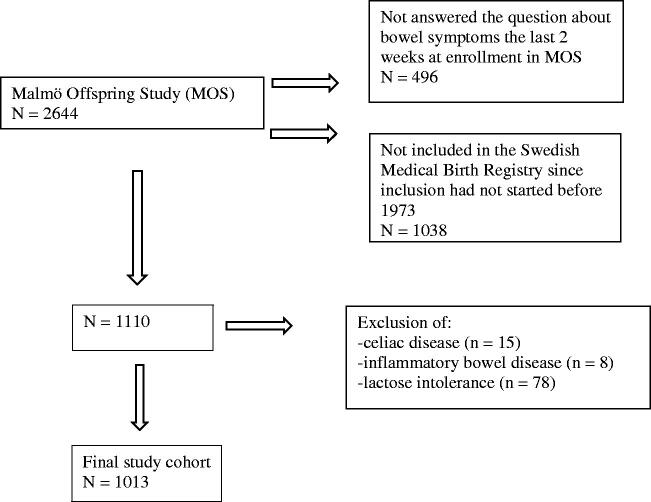
Flow chart over the inclusion process in Malmö Offspring Study.

This is a population-based study combining data collected from the Swedish Medical Birth Registry (MBR) with the Malmö Offspring Study (MOS) (Figure 1) [18,19]. Participants in MOS completed questionnaires regarding bowel symptoms in the form of the visual analog scale for irritable bowel syndrome (VAS-IBS) as well as information concerning their socioeconomic background, lifestyle habits, and health-related questions. Perinatal data for the participants were retrieved from the MBR. The primary outcome was to estimate the risk of developing bowel symptoms later in life when born with low gestational age, LBW, low Apgar score or SGA. The secondary outcomes were to estimate the risk of severe specific bowel symptoms. The odds ratios were calculated using the logistic regression model.

### The Swedish Medical Birth Registry

The Swedish MBR was established in 1973 to provide a complete data set for obstetric care on a national scale. It contains data from more than 98% of the pregnancies that have resulted in childbirth in Sweden since start. It contains information regarding pregnancy, delivery, and the newborn child, along with data concerning the mother’s health status, all recorded by health personnel [20].

### The Malmö Offspring Study

The MOS is an ongoing population-based cohort (*n* = 2644), with the single inclusion criteria of being either the adult children or grandchildren of the participants in the Malmö Diet and Cancer – Cardiovascular (MDC-CV) cohort [18]. The MDC-CV itself was formed by 6103 randomly selected individuals from the original study called the Malmö Diet and Cancer Study (MDCS). MDCS was formed between 1991 and 1996, and its 28,098 participants were born between the years of 1923 and 1950 [19]. The MOS data for this study were attained between 2013 and 2017 [18]. At inclusion in MOS, age, sex, and body mass index (BMI) were registered. A questionnaire regarding educational level, occupation, marital status, smoking habits, alcohol habits, physical activity, stress level, and medical history was completed. To assess self-reported IBS related to the Rome III criteria [18,21], the participants had to answer the question ‘Have you several times during a month suffered from abdominal pain related to irregular bowel habits, which is called IBS?’ If they answered yes, they were referred to as having self-reported IBS.

### Study participants

All 2644 MOS participants (46.7% participation rate) who had accepted their invitations until 2017 and had answered the question on whether they had experienced bowel symptoms during the past 2 weeks were initially included. Not answering that specific question equaled exclusion from the study (*n* = 496). Participants born before the start of the Swedish MBR (1973) were excluded, since no perinatal data were then available (*n* = 1038). Participants who reported being diagnosed with organic bowel diseases or characteristics such as celiac disease (*n* = 15), inflammatory bowel diseases (*n* = 8), and/or lactose intolerance (*n* = 78) were also excluded, rendering 1013 participants (mean age: 29.01 ± 6.77 years; range: 18.35–44.04 years) that formed the final study population (Figure 1).

### Visual analog scale for irritable bowel syndrome

VAS-IBS is a validated questionnaire measuring functional symptoms of abdominal pain, diarrhea, constipation, bloating and flatulence, vomiting and nausea, intestinal symptoms’ influence on daily life and psychological well-being in patients with IBS [22]. The participants that answered yes to the question regarding whether they had experienced bowel symptoms during the past 2 weeks were encouraged to grade their symptoms on a scale where 0 represents no symptoms and 100 mm indicates maximal symptoms or influence of symptoms. This questionnaire is an acceptable homogeneous patient-reported questionnaire with a Cronbach’s alpha internal consistency reliability coefficient of 0.85. The scales were inverted from the original publication [22].

### Data categorization

Educational level was stratified into completed elementary school maximum 9 years, upper secondary school, and university degree. Smoking habits were divided into current smokers and non-smokers/ex-smokers. The alcohol intake was calculated into grams of alcohol/week. Occupation was stratified into employed at the study time point or student, unemployed or others. Marital status was divided into living alone, married, and cohabiting or other. Mental stress level was divided into two categories, yes or no, where ‘yes’ indicated self-perceived constant stress during the past 12 months. The amount of physical activity in the leisure time during the past 12 months were categorized into sedentary (mostly stationary activities, less than 2 h of physical activity per week), moderate (at least 2 h of physical activity per week, usually without breaking a sweat, for example walking), regularly but moderate (exercising enough to break a sweat, e.g. running, for at least 30 min 1–2 times a week) and exercising regularly (on average physical activities for at least 30 min, such as running, minimum three times a weeks). The early life exposures were divided by the commonly used categorization. Birth weight was split into two categories: ≥2500 g (normal birth weight) and <2500 g (LBW) [9,11]. Gestational age was divided into ≥37 weeks (term) and <37 weeks (preterm) [8]. Apgar score (range 0–10) was split into ≥7 and <7 [17]. SGA was classified as birth weight in relation to gestational age in the 10th percentile of the population [14].

### Statistical analyses

This study assessed the following research questions: (1) Is preterm birth, LBW, low Apgar score, or SGA associated with the development of bowel symptoms? (2) Is there an association between these early life factors and severity of specific bowel symptoms?

Age, BMI, gestational age, and birth weight were normally distributed, whereas other variables were non-normally distributed according to visual inspection of histograms. Comparisons of continuous variables were therefore calculated by the Mann–Whitney *U* test or Student *t* test. Categorical variables were calculated by the Chi-square test.

The logistic regression model was used to estimate odds ratio (OR) and 95% confidence interval (CI) of (1) functional bowel symptoms past 2 weeks and (2) self-reported IBS as defined outcomes, with the dichotomous variables (0/1), and the perinatal data used as independent variables: gestational age, birth weight, Apgar score, and SGA. Gestational age and birth weight were calculated both as dichotomous values of preterm/term and LBW/NBW, respectively, and as continuous values. The median value of each functional symptom was used as a cut-off, creating one group with a small amount of symptom and a second group with a high amount of symptom. Logistic regression was used to compare participants with a high burden of symptoms with the control group without any bowel symptoms at all, in relation to perinatal factors. The full model was adjusted for sex and self-reported constant stress during the past year, since these were the covariates found to be significantly different between participants with or without bowel symptoms, whereas sex, self-reported constant stress during the past year, smoking and alcohol intake were adjusted for in the calculations between self-reported IBS and participants without IBS. Descriptive data are presented as mean ± standard deviation or median and interquartile range (IQR) for the continuous variables and number and percentage for categorical variables. *p* < 0.05 was considered statistically significant. Calculations were performed in SPSS (version 26.0; IBM Corporation, Armonk, NY).

## Results

### Population characteristics

In total, 1013 participants were included in the final study population. Of these, 253 participants (25.0%) had experienced bowel symptoms during the past 2 weeks (cases) whereas 760 (75.0%) participants did not (controls). There was a significant association between having experienced bowel symptoms during the past 2 weeks and having a self-reported diagnosis of IBS (*n* = 179) (*p* < 0.001). Out of the 253 participants with bowel symptoms during the past 2 weeks, 53.4% reported they were suffering from IBS. Among control participants, only 5.9% had been diagnosed with IBS.

More women than men suffered from symptoms or self-reported IBS (*p* < 0.001). The median age and median BMI were similar in all groups. There was no difference between the group with or without symptoms regarding socioeconomic data, smoking, alcohol habits or physical activity, whereas smoking and higher alcohol intake was more prevalent in participants with self-reported IBS. Constant stress during the past 12 months was significantly more commonly reported among cases than controls (65.2% versus 46.3%) (Table 1), as well as poorer psychological well-being (Table 2). More participants with self-reported IBS experienced chronic stress during the last year (73.7% versus 53.4%), and all bowel symptoms were aggravated in the IBS group. Both gestational age (*p* = 0.091) and Apgar score (*p* = 0.063) tended to be lower in the group with bowel symptoms, whereas birth weight was slightly lower in the group with self-reported IBS (*p* = 0.063), compared with the controls, although not reaching statistical significance. The most prominent bowel symptom was bloating and flatulence, followed by intestinal symptoms’ influence on daily life, diarrhea, and abdominal pain (Table 2).

**Table 1.** Study population characteristics.

Characteristics	Bowel symptoms (*n* = 253)	No symptoms (*n* = 760)	*p*-value	Self-reported IBS (*n* = 179)	No IBS (*n* = 832)	*p*-value
Sex (*n*, %)			<0.001			<0.001
Women	169 (66.8)	377 (49.6)		124 (69.3)	421 (50.6)	
Men	84 (33.2)	383 (50.4)		55 (30.7)	411 (49.4)	
Age (years)	28.72 ± 6.29	29.11 ± 6.93	0.436	29.50 ± 6.52	28.90 ± 6.82	0.268
Body Mass Index (kg/m^2^)	25.04 ± 4.57	24.87 ± 4.42	0.593	25.12 ± 4.52	24.88 ± 4.45	0.506
Education (*n*, %)			0.532			0.963
Completed elementary school	9 (3.6)	33 (4.3)		8 (4.5)	34 (4.1)	
Upper secondary school	139 (54.9)	441 (58.0)		102 (57.0)	477 (57.3)	
University/college graduate	103 (40.7)	282 (37.1)		67 (37.4)	317 (38.1)	
*Missing data*	2 (0.8)	4 (0.5)		2 (1.1)	4 (0.5)	
Occupation (*n*, %)			0.130			0.573
Employed	165 (65.2)	534 (70.3)		118 (65.9)	579 (69.6)	
Student, unemployed, other	80 (31.6)	193 (25.4)		54 (30.2)	219 (26.3)	
*Missing data*	8 (3.2)	33 (4.3)		7 (3.9)	34 (4.1)	
Marital status (*n*, %)			0.867			0.736
Unmarried and living alone	66 (26.1)	210 (27.6)		46 (25.7)	230 (27.6)	
Married and cohabiting	145 (57.3)	424 (55.8)		104 (58.1)	460 (55.3)	
Other	41(16.2)	124 (16.4)		27 (15.1)	139 (16.7)	
*Missing data*	1 (0.4)	2 (0.2)		2 (1.1)	3 (0.4)	
Smoking habits (*n*, %)			0.112			0.042
Never smoked/ex-smoker	199 (78.7)	629 (82.8)		137 (76.5)	689 (82.8)	
Smoker	54 (21.3)	128 (16.8)		42 (23.5)	140 (16.8)	
*Missing data*	0	3 (0.4)		0	3 (0.4)	
Alcohol intake (g/week)	15 (11-45)	24 (11-50)	0.323	14 (4-45)	24 (10-50)	0.040
*Missing data*	1 (0.4)	4 (0.05)		1 (0.1)	4 (0.5)	
Physical activity past 12 months (*n*, %)			0.128			0.260
Sedentary	26 (10.3)	65 (8.6)		20 (11.2)	71 (8.5)	
Moderate	81 (32.0)	257 (33.8)		61 (34.1)	276 (33.2)	
Moderate, but regular	78 (30.8)	196 (25.8)		53 (34.1)	221 (26.6)	
Training regularly	65 (25.7)	236 (31.1)		43 (24.0)	257 (30.9)	
*Missing data*	3 (1.2)	6 (0.8)		2 (1.1)	7 (0.8)	
Constant stress past 12 months (*n*, %)			<0.001			<0.001
Yes	165 (65.2)	353 (46.3)		132 (73.7)	444 (53.4)	
No	88 (34.8)	404 (53.2)		47 (26.3)	384 (46.2)	
*Missing data*	0	4 (0.5)		0	4 (0.5)	

There were two missing values of self-reported irritable bowel syndrome (IBS). Values are presented as mean ± standard deviation, median (interquartile range) and numbers and percentages. Mann–Whitney *U* test, Student *t* test, or Chi-square test. A *p*-value <0.05 was considered statistically significant.

**Table 2.** Early life exposures and bowel symptoms in the study cohort.

	Bowel symptoms (*n* = 253)	No symptoms (*n* = 760)	*p*-value	Self-reported IBS (*n* = 179)	No IBS (*n* = 832)	*p*-value
Self-reported irritable bowel syndrome	135 (53.4)	44 (5.9)	<0.001			
*Missing data*	1 (0.4)	1 (0.1)				
Early life exposures						
Gestational age (weeks)	39.16 ± 2.01	39.42 ± 2.01	0.091	39.16 ± 2.02	39.39 ± 2.01	0.173
*Missing data*	13 (5.1)	36 (4.7)		7 (3.9)	42 (5.0)	
Birth weight (g)	3423 ± 618	3488 ± 587	0.140	3394 ± 580	3486 ± 597	0.063
*Missing data*	8 (3.2)	27 (3.6)		5 (2.8)	30 (3.6)	
APGAR-score						
1 min after birth	9 (9-9)	9 (9-9)	0.063	9 (9-9)	9 (9-9)	0.283
*Missing data*	9 (3.6)	36 (4.7)		5 (2.8)	40 (4.8)	
5 min after birth	10 (10-10)	10 (10-10)	0.804			0.877
*Missing data*	43 (17.0)	167 (22.0)		32 (17.9)	178 (21.4)	
Small for gestational age	11 (4.3)	22 (2.9)	0.287	8 (4.5)	25 (3.0)	0.359
*Missing data*	19 (7.5)	73 (9.6)		12 (6.7)	80 (9.6)	
Bowel symptoms (mm)						
Abdominal pain	27 (10-58)	–		45 (17-62)	12 (1-30)	<0.001
*Missing data*	20 (7.9)			54 (30.2)	710 (85.3)	
Diarrhea	30 (5-58)	–		37 (15-64)	13 (0-48)	<0.001
*Missing data*	24 (9.5)			58 (32.8)	711 (85.5)	
Constipation	10 (1-55)	–		30 (2-66)	4 (0-23)	<0.001
*Missing data*	31 (12.3)			64 (35.8)	712 (85.6)	
Bloating and flatulence	54 (22-72)	–		66 (40-79)	24 (4-60)	<0.001
*Missing data*	20 (7.9)			54 (30.2)	711 (85.5)	
Vomiting and nausea	5 (0-50)	–		13 (0-55)	3 (0-21)	0.030
*Missing data*	39 (15.4)			69 (38.5)	715 (85.9)	
Symptoms influence on daily life	34 (10-61)	–		51 (20-75)	16 (1-48)	<0.001
*Missing data*	14 (5.5)			52 (29.1)	707 (85.0)	
Psychological well-being	25 (10-56)	18 (6-30)	<0.001	26 (10-60)	18 (6-33)	<0.001
*Missing data*	9 (3.6)	58 (7.6)		7 (3.9)	60 (7.2)	

There were two missing values of self-reported irritable bowel syndrome (IBS). Apgar score ranges between 0 and 10, with higher values indicating higher alertness and better health at birth [16, 17]. Small for gestational age (SGA) was defined as a birth weight in relation to gestational age in the 10^th^ percentile of the population [14]. Symptoms were measured on the visual analog scale for irritable bowel syndrome (VAS-IBS) in mm, where 0 represents no symptoms and 100 represents maximal symptoms during the past 2 weeks [22]. Values are presented as mean ± standard deviation, median (interquartile range) and numbers and percentages. Mann–Whitney *U* test, Student *t* test, or Chi-square test. A *p*-value <0.05 was considered statistically significant.

### Relations between early life factor exposures and adult bowel symptoms

There was a non-significant trend for an association between being born preterm and bowel symptoms, in comparison to born at term (OR: 1.667; 95% CI: 0.940–2.955; *p* = 0.080), which was also found when calculating the gestational age used as a continuous variable (*p* = 0.085) (Table 3). Further calculations stratifying for severity did not show any significant associations between prematurity (preterm) and severe bowel symptoms (Supplementary Table 1). Neither LBW or low Apgar score nor SGA had any significant associations with functional bowel symptoms (Table 3). Early life factors did not show any associations with self-reported IBS (data not shown).

**Table 3.** Odds ratio of associations between early life exposures and reported bowel symptoms during the past 2 weeks.

Exposures	Bowel symptoms		Crude model			Full model		
	Symptoms	No symptoms	OR	95% CI	*p*-value	OR	95% CI	*p*-value
Gestational age (weeks)								
≥37	220 (87.0)	681 (89.6)	1.00			1.00		
<37	20 (7.9)	43 (5.7)	1.440	0.829–2.500	0.195	1.667	0.940–2.955	0.080
P continuous values					0.093			0.085
Birth weight (g)								
≥2500	227 (89.7)	696 (91.6)	1.00			1.00		
<2500	18 (7.1)	37 (4.9)	1.581	0.878–2.847	0.127	1.619	0.881–2.975	0.120
P continuous values					0.141			0.298
Apgar scores								
1 min after birth								
≥7	230 (90.9)	695 (91.4)	1.00			1.00		
<7	14 (5.5)	29 (3.8)	1.459	0.758–2.808	0.259	1.494	0.756–2.951	0.248
5 min after birth								
≥7	209 (82.6)	588 (77.4)	1.00			1.00		
<7	1 (0.4)	5 (0.7)	0.563	0.065–4.844	0.601	0.774	0.087–6.846	0.817
SGA								
No	223 (88.1)	665 (87.5)	1.00			1.00		
Yes	11 (4.3)	22 (2.9)	1.491	0.712-3.123	0.290	1.565	0.726-3.374	0.253

OR: odds ratio; CI: Confidence interval. Apgar score ranges between 0 and 10, with higher values indicating higher alertness and better health at birth [16, 17]. Small for gestational age (SGA) was defined as a birth weight in the 10th percentile [14]. Symptoms were measured on the visual analog scale for irritable bowel syndrome (VAS-IBS) in mm, where 0 represents no symptoms and 100 represents maximal symptoms during the past 2 weeks [22]. Values are presented as numbers and percentages. Logistic regression model adjusted for sex and chronic mental stress. A *p*-value <0.05 was considered statistically significant.

Both LBW (*p* = 0.038) and SGA (*p* = 0.043), in contrast to low Apgar score, were associated with a high degree of the symptom´s influence on daily life (Table 4) but did not associate with severity of specific bowel symptoms (data not shown).

**Table 4.** Odds ratio of associations between early life exposures and reported moderate to severe intestinal symptoms´ influence on daily life during the past 2 weeks.

Exposures	Symptoms’ influence on daily life	No influence on daily life	Crude model			Full model		
			OR	95% CI	*p*-value	OR	95% CI	*p*-value
Birth weight (g)								
≥2500	107 (88.4)	696 (91.6)	1.00			1.00		
<2500	9 (7.4)	37 (4.9)	1.677	0.784–3.588	0.182	1.711	0.767–3.814	0.189
P continuous values					0.011			0.038
Apgar scores								
≥7	111 (91.7)	695 (91.4)	1.00			1.00		
<7	5 (4.1)	29 (3.8)	1.080	0.409–2.848	0.877	1.218	0.440–3.373	0.704
SGA								
No	102 (84.3)	695 (96.9)	1.00			1.00		
Yes	8 (6.6)	22 (3.1)	2.371	1.028–5.467	0.043	2.543	1.031–6.271	0.043

OR: odds ratio; CI: Confidence interval; SGA: small for gestational age. Apgar score (measured after 1 min) ranges between 0 and 10, with higher values indicating higher alertness and better health at birth [16, 17]. SGA was defined as a birth weight in the 10th percentile [14]. Symptoms were all self-reported and measured on the visual analog scale for irritable bowel syndrome (VAS-IBS) in mm, where 0 represents no symptoms and 100 represents maximal symptoms during the past 2 weeks [22]. Intestinal symptoms’ influence on daily life was classified as values above the median value and compared with asymptomatic controls. Prevalence of intestinal symptoms´ influence on daily life (yes/no) are presented as numbers and percentages. Logistic regression model adjusted for sex and chronic mental stress. A *p*-value <0.05 was considered statistically significant.

## Discussion

### Statement of principal findings

The main finding of this observational study was that female sex and experience of chronic mental stress were much more prevalent in participants with functional bowel symptoms and self-reported IBS, compared with non-symptomatic controls. Lower gestational age tended to be associated with a higher risk of developing functional bowel symptoms later in life, whereas other early life factors were not associated with bowel symptoms or self-reported IBS. Lower birth weight and SGA were not associated with the presence of bowel symptoms but were significantly associated with the negative influence of bowel symptoms on daily life.

### Strengths and weaknesses of the study

The strength of the present population-based study is the large sample of participants from outside of specialist healthcare and the focus on associations between early life factors and individual bowel symptoms and their severity. The observational design limits the analysis of causality of our findings. Several calculations showed trends and non-significant differences between groups, which may depend on several missing values reducing the sample size. It is difficult to evaluate if these trends had disappeared or had turned significant whether the sample size had been larger. Another limitation is the lack of detailed data regarding perinatal stress factors such as mode of delivery, neonatal respiratory distress, necrotizing enterocolitis, or food allergy. However, all subjects with organic bowel disease in adulthood, celiac disease, or lactose intolerance were excluded, which are the most common food intolerances to be found in the community. The diagnosis of IBS was assessed from self-report and not the complete Rome IV questionnaire, which should have added too many questions for a population-based study. Several additional confounders could be of importance for the development of bowel symptoms, for example dietary factors [2], which were not added in the full model.

### Findings in relation to other studies

Previous studies have described an increased risk for FBD in subjects born with LBW [12,13,23]. Bengtson et al. [23] found birth weight below 1500 g to be a significant contributor to the development of IBS. Since we had very few participants in this low birth weight category, we could not perform such calculations. On the other hand, Olén et al. [10] found a lower gestational age to be associated with a decreased risk of IBS in young adulthood. Study designs and study populations differ vastly, one of the main differences being the diagnostic criteria for IBS and functional diseases, which varies over time and thereby complicates comparisons between studies [24]. We observed that 25.0% of our relatively young study population suffered from functional bowel symptoms, and 17.7% suffered from self-reported IBS according to the Rome III criteria. Other studies reported 5.3% self-assed IBS according to Rome II criteria [23], 1.3% IBS diagnosed through specialist care (Rome 1-III) [13] and 1.1% IBS prevalence in subjects included from hospital-based outpatient care (Rome II-III) [10]. Therefore, individual assessment of functional bowel symptoms is preferable, since we then can compare study cohorts over time independent of varying diagnosis criteria [1,21], and also since contradictory associations of different bowel symptoms may disturb the conclusions [18]. Study cohorts which represent a selected hospital population probably have different underlying pathological mechanisms to the heterogeneous IBS disease compared with study cohorts from the general population [25]. Most of the patients are cared for in primary healthcare centers, and less than half of individuals suffering from IBS seek healthcare, making the study design of the present population-based study more suitable for evaluating this disease entity in the population at large [26]. We cannot exclude the possibility that lower birth weight is in fact associated with functional bowel symptoms in adulthood, based on our study sample. However, the current results suggest that it is the experience of symptoms and their influence on daily life, rather than the presence of symptoms, which is associated with early life factors. The question concerning how the symptoms influence daily life is seldom asked, and therefore, this information is often not available.

FBD may reflect several different etiologies, where brain–gut interaction, bowel dysmotility, immune activations, and visceral hypersensitivity may contribute to the symptoms [2]. In the bidirectional communication between the gut and brain, the brain plays an essential role in how much of this information from the gut that is consciously perceived (visceral sensitivity) [6]. The central processing of gut-related information involves a coping strategy, which strongly correlates with the severity of pain symptoms experienced [2]. The term sensitization refers to an over-excitability following nociceptive stimuli in peripheral and/or central neurons, which causes pain in subjects exposed to normal stimuli [27]. The subjective pain linked to IBS is thought to be sustained both from peripheral impulses from the colon as well as from central sensitizing [5,6]. The co-morbidity of IBS and psychological distress is common [4], and the two conditions also exhibit a strong correlation between the severity of IBS symptoms and psychiatric disorders [5].

Major life traumas increase the risk to develop IBS, but there are conflicting results to what degree stressful life events can exacerbate IBS symptoms [5]. Animal models have shown bowel irritation in newborns to result in chronic visceral hypersensitivity associated with central sensitization in the absence of peripheral pathology [28]. In addition, unpredictable early life trauma has been shown to yield colonic hypersensitivity in adulthood [29]. The increased likelihood of medical interventions among preterm and LBW neonates, along with a presumed heightened stress level solely from being born after a shorter gestational period or with lower birth weight, may influence the development of visceral hypersensitivity. The increased impact on intestinal symptom´s on daily life observed in the current study in subjects with LBW and SGA could be related to impaired coping mechanisms and central sensitizing.

In addition to neurons, glial cells, interstitial cells of Cajal, and telocytes have been discovered to play an essential part in the enteric nervous system (ENS) function [30]. Gestational age below the full 40 weeks and low birth weights could hypothetically affect the maturation of ENS, and thereby lead to peripheral sensitization [8]. Significant improvements in perinatal care have been made during recent decades, leading to a substantial increase in the survival of infants born preterm with low birth weight. With these great advances, concerns regarding the long-term effects of being born with immature organs have also emerged [9]. Even births after 37–39 weeks of gestation carry a relatively worse outcome risk compared to births after 40 weeks of gestation [8]. On top of the postnatal development of organs, there is also the side effects of intensive care unit interventions to consider, such as suboptimal nutrition, invasive procedures, gastric suction, and side effects from medication [8].

### Meaning of the study

Our results suggest that the effect of early life factors is moderate on the reporting of bowel symptoms in adulthood. However, an association between LBW and SGA, on the one hand, and enhanced influence of intestinal symptoms on daily life, on the other hand, was observed. Further studies are needed to elucidate on the role of early life factors in relation to functional disorders in adult life.

## Supplementary Material

Supplemental Material
