# Supplementary material for: A possible association between early life factors and burden of functional bowel symptoms in adulthood
Source: Scand J Prim Health Care. 2021 Nov 22;39(4):506–14. doi: 10.1080/02813432.2021.2004823 (PMC8725981; doi:10.1080/02813432.2021.2004823)
Supplement: Supplemental Material [file IPRI_A_2004823_SM4834.docx]

**Supplementary Table 1.** Odds ratio of reported associations between preterm birth and moderate to severe specific symptoms during the past 2 weeks

| **Gestational age** (weeks) | **Symptoms** | | **Crude model** | | | **Full model** | | |
| --- | --- | --- | --- | --- | --- | --- | --- | --- |
|  | **Abdominal pain** | **No pain** | **OR** | **95% CI** | **p-value** | **OR** | **95% CI** | **p-value** |
| ≥37 | 104 (88.9) | 681 (89.6) | 1.00 |  |  | 1.00 |  |  |
| <37 | 7 (6.0) | 43 (578) | 1.066 | 0.467-2.433 | 0.879 | 1.280 | 0.536-3.056 | 0.578 |
| P continuous value |  |  |  |  | 0.095 |  |  | 0.106 |
|  | **Diarrhea** | **No diarrhea** |  |  |  |  |  |  |
| ≥37 | 101 (87.8) | 681 (89.6) | 1.00 |  |  | 1.00 |  |  |
| <37 | 7 (6.1) | 43 (5.7) | 1.198 | 0.481-2.506 | 0.825 | 1.295 | 0.553-3.031 | 0.552 |
| P continuous value |  |  |  |  | 0.651 |  |  | 0.640 |
|  | **Constipation** | **No constipation** |  |  |  |  |  |  |
| ≥37 | 97 (87.4) | 681 (89.6) | 1.00 |  |  | 1.00 |  |  |
| <37 | 9 (8.1) | 43 (5.7) | 1.469 | 0.695-3.109 | 0.314 | 1.738 | 0.792-3.813 | 0.168 |
| P continuous value |  |  |  |  | 0.190 |  |  | 0.234 |
|  | **Bloating and flatulence** | **No Bloating**  **and flatulence** |  |  |  |  |  |  |
| ≥37 | 103 (87.3) | 681 (89.6) | 1.00 |  |  | 1.00 |  |  |
| <37 | 8 (6.8) | 43 (5.7) | 1.230 | 0.562-2.690 | 0.604 | 1.475 | 0.646-3.369 | 0.356 |
| P continuous value |  |  |  |  | 0.559 |  |  | 0.586 |
|  | **Vomiting and nausea** | **No vomiting** |  |  |  |  |  |  |
| ≥37 | 97 (87.4) | 681 (89.6) | 1.00 |  |  | 1.00 |  |  |
| <37 | 8 (7.2) | 43 (5.7) | 1.306 | 0.596-2.861 | 0.504 | 1.563 | 0.686-3.563 | 0.288 |
| P continuous value |  |  |  |  |  | 0.283 |  | 0.345 |
|  | **Symptoms´ influence on daily life** | **No influence** |  |  |  |  |  |  |
| ≥37 | 106 (87.6) | 681 (89.6) | 1.00 |  |  | 1.00 |  |  |
| <37 | 9 (7.4) | 43 (5.7) | 1.345 | 0.637-2.838 | 0.437 | 1.624 | 0.737-3.576 | 0.229 |
| P continuous value |  |  |  |  | 0.136 |  |  | 0.134 |

OR = odds ratio, CI = Confidence interval. Symptoms were measured on the visual analog scale for irritable bowel syndrome (VAS-IBS) in mm, where 0 represents no symptoms and 100 represents maximal symptoms during the past 2 weeks [22]. Symptoms were classified as values above the median value and compared with asymptomatic controls. Prevalence of symptoms (yes/no) are presented as numbers and percentages. Logistic regression model adjusted for sex and chronic mental stress. A p-value <0.05 was considered statistically significant.
